# Oncogenic role of TWF2 in human tumors: A pan-cancer analysis

**DOI:** 10.1515/med-2022-0547

**Published:** 2022-09-05

**Authors:** Wenjie Liu, Gengwei Huo, Peng Chen

**Affiliations:** Department of Thoracic Oncology, Tianjin Medical University Cancer Institute and Hospital, National Clinical Research Center for Cancer, Key Laboratory of Cancer Prevention and Therapy of Tianjin, Tianjin’s Clinical Research Center for Cancer, Tianjin, 300060, China; Department of Thoracic Oncology, Tianjin Medical University Cancer Institute and Hospital, National Clinical Research Center for Cancer, Key Laboratory of Cancer Prevention and Therapy of Tianjin, Tianjin, China; Tianjin’s Clinical Research Center for Cancer, Tianjin, 300060, China; Department of Oncology, Jining No. 1 People’s Hospital, Jining, 272000, Shandong, China

**Keywords:** TWF2, cancer, survival, prognosis, immune infiltration

## Abstract

To develop effective medicines, researchers must first understand the common and distinct mechanisms that drive oncogenic processes in human cancers. TWF1 and TWF2 belong to the actin-depolymerizing factor homology family. TWF1 has been identified as an important gene in lung, breast, and pancreatic cancer in recent investigations. TWF2’s role in cancer remains largely unknown, no comprehensive pan-cancer studies have been conducted. We utilized the The Cancer Genome Atlas and Gene Expression Omnibus datasets to investigate the role of TWF2 in different types of cancers. TWF2 transcription in cancers and the number of TWF2 mutations were examined as part of our study. We also examined the possible functional pathways involved in TWF2-mediated oncogenicity. Our pan-cancer analysis provided a complete overview of the oncogenic effects of TWF2 in a wide range of human malignancies.

## 1 Introduction

To gain a deeper understanding of the complex process of cancer formation, it is necessary to recognize and characterize new pan-cancer genes. The Cancer Genome Atlas (TCGA) and the Gene Expression Omnibus (GEO) contain a substantial amount of cancer-related functional genomic datasets from various cancer types that can be utilized for pan-cancer analysis [1,2,3] (Table 1).

TWF (Twinfilin), a protein that regulates actin dynamics, is an evolutionarily conserved protein with two Actin-Depolymerizing Factor Homology domains [4]. TWF proteins bind to actin monomers and heterodimeric capping proteins [5,6]. TWF1 (Twinfilin Actin Binding Protein 1) and TWF2 (Twinfilin Actin Binding Protein 2) showed different tissue distributions in mammals, and initial studies have indicated that TWF1 was the major isoform in the developing embryo and non-muscle tissues in most adult mouse, whereas TWF2 was mainly expressed in heart, skeletal muscle, and spleen [7]. Recent research studies have suggested that TWF1 was highly expressed in various solid tumors and may be regarded as an important gene in lung, pancreas, and breast cancers [8,9,10,11]. It has previously been demonstrated that the transcription of TWF1 in LUAD tissues is linked to a poorer TNM stage, more lymph node metastases, a larger tumor size, and late clinical staging, among other factors [9]. Homologous to the actin depolymerizing factor (ADF) as a member of the ADF homology family, TWF2 is a protein with two ADF-homology domains. A6RP, A6r, or PTK9L may alternatively be referred to as TWF2. TWF2’s role in cancer development, on the other hand, remained a mystery.

The transcription profile of TWF2 was investigated in a pan-cancer analysis using data from TCGA and GEO databases. When comparing TWF2 transcription profiles across different types of cancers, the survival status, genetic alterations, and essential biological pathways were all considered. The results of this comprehensive analysis suggest that TWF2 may play a role in the pathogenesis and prognosis of a wide spectrum of malignancies.

## 2 Materials and methods

### 2.1 Gene mapping analysis

TWF2 genome location information was acquired from the UCSC (http://genome.ucsc.edu/) genome browser [12]. The National Center for Biotechnology Information (https://ncbi.nlm.nih.gov/) conducted a conserved functional domain analysis of TWF2 in diverse species.

### 2.2 HPA-gene transcription analysis

The transcription levels of TWF2 under physiological conditions in different cell and tissue types were analyzed using the HPA (https://www.proteinatlas.org) (Human Protein Atlas) database. The internal normalization pipeline was used when combining the HPA and Genotype-Tissue Expression (GTEx) transcriptomics datasets. This consensus dataset consists of normalized expression (nTPM) levels for 55 tissue types.

### 2.3 Gene transcription analysis

In our study, Tumour Immune Estimation Resource 2 (TIMER2) was used to analyze the transcription profile of TWF2 in tumors and adjacent normal tissues. For tumors that lack or contain just a small amount of healthy tissue, we utilized the Gene Expression Profiling Interactive Analysis 2 (GEPIA2) tool to generate box plots from GTEx databases using the GTEx databases, with a *p*-value threshold of 0.01, with a fold change of log_2_FC cutoff of one, and “Match TCGA healthy and GTEx data.” All TCGA tumors were analyzed using the HEPIA2 program for TWF2 transcription analysis. Violin and box plots were created using translated expression data log2 [transcripts per million + 1].

The UALCAN (http://ualcan.path.uab.edu) databases were utilized to evaluate tumor omics data and to undertake protein transcription analysis from CPTAC (Clinical Proteomic Tumor Analysis Consortium) databases, which we found to be very useful. A two-tailed *p* value of less than 0.05 was considered statistically significant [13]. *p < 0.05; **p < 0.01; ***p < 0.001.

### 2.4 Survival prognosis analysis

All TCGA tumors were utilized to construct TWF2 survival maps and survival plots to assess Disease-Free Survival (DFS) and Overall Survival (OS). To divide the transcription cohorts into low- and high-expression cohorts, a cut-off (50 percent) was utilized [14]. Log-rank tests were used to analyze the validity of our hypotheses. The Kaplan–Meier plotter was utilized to pool many GEO databases that analyze the Post Progression Survival (PPS), Progression-Free Survival (PFS), First Progression (FP), and OS (http://kmplot.com/analysis/). High and low transcription groups were identified using the “auto-selection of the best cutoff” function and calculated the HR, CI, and log-rank *p*-values for our study.

### 2.5 Genetic alteration analysis

We collected mutant site information, mutation type, mutation frequency, and Copy Number Alteration (CNA) from all TCGA malignancies using the cBioPortal (https://www.cbioportal.org/) databases.

### 2.6 Correlation of TWF2 and MSI/TMB

Analysis of TWF2 transcription and Microsatellite Instability (MSI) or Tumour Mutational Burden (TMB) in TCGA tumors was performed using the web of “http://sangerbox.com/Tool” [15]. Spearman’s rank correlation tests were used to obtain the *p* value and partial correlation value.

### 2.7 Immune infiltration analysis

TWF2 transcription and immune infiltrates were examined in all TCGA tumors using the TIMER2 program. We focused on malignancy-related fibroblasts, neutrophils, T-cell regulatory cells, and endothelial cells. Estimates were made using TIMER, CIBERSORT-ABS, CIBERSORT, QUANTISEQ, EPIC, MCPCOUNTER, XCELL, and TIDE algorithms. The purity-adjusted Spearman’s rank correlation test was used to calculate the *p*-value and partial correlation (cor) values.

### 2.8 TWF2-related gene enrichment analysis

The interactome network was further analyzed using the STRING website (https://cn.string-db.org/) to determine TWF2 binding proteins by available experiments. We chose the significance of network edges (“evidence”), the active interaction sources (“experiments”), the maximum number of participants to display (“no more than 50 interactors”), and the minimal interaction score [“Medium confidence (0.400)”] to reduce the bias.

In order to extract the 100 highest TWF2-related genes from TCGA cancer and healthy tissue datasets, the program GEPIA2 was employed. In the subsequent stage, we determined the relationship between TWF2 and the target genes that we previously identified using Pearson correlation analysis. All statistical significance values (*p-*value and correlation coefficient) were computed and presented in the appropriate plot panels for each of the variables. *p*-Value and partially correlated from the purity-adjusted Spearman’s rank correlation test are displayed as heatmaps of the transcription patterns for the selected genes. GO|KEGG (Gene Ontology | Kyoto Encyclopedia of Genes and Genomes) enrichment and pathway analysis were done by integrating and filtering two sets of data. The BP, CC, and MF, together with KEGG pathway analysis, were visualized using the R packages “clusterProfiler” and “ggplot2” R project software (https://www.r-project.org/) (version 3.6.3) in this investigation [16].

## 3 Results

### 3.1 Gene transcription analysis

**Figure A1:**
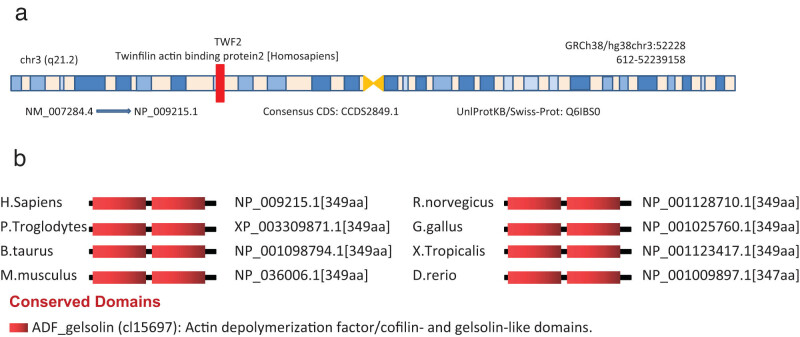
TWF2 structural properties in several species. (a) The hg38 genome harbours the human TWF2 gene; (b) TWF2’s conserved protein domains in different species.

Human TWF2 (NM 007284.4 mRNA or NP 009215.1 protein, Figure A1a) was the focus of this study because of its potential oncogenesis. Figure A1b shows that the ADF gelsolin (cl15697) domain is present in all TWF2 proteins from diverse species, including those from humans and other primate species, such as pan troglodytes, homo sapiens, and bos taurus.

**Figure A2:**
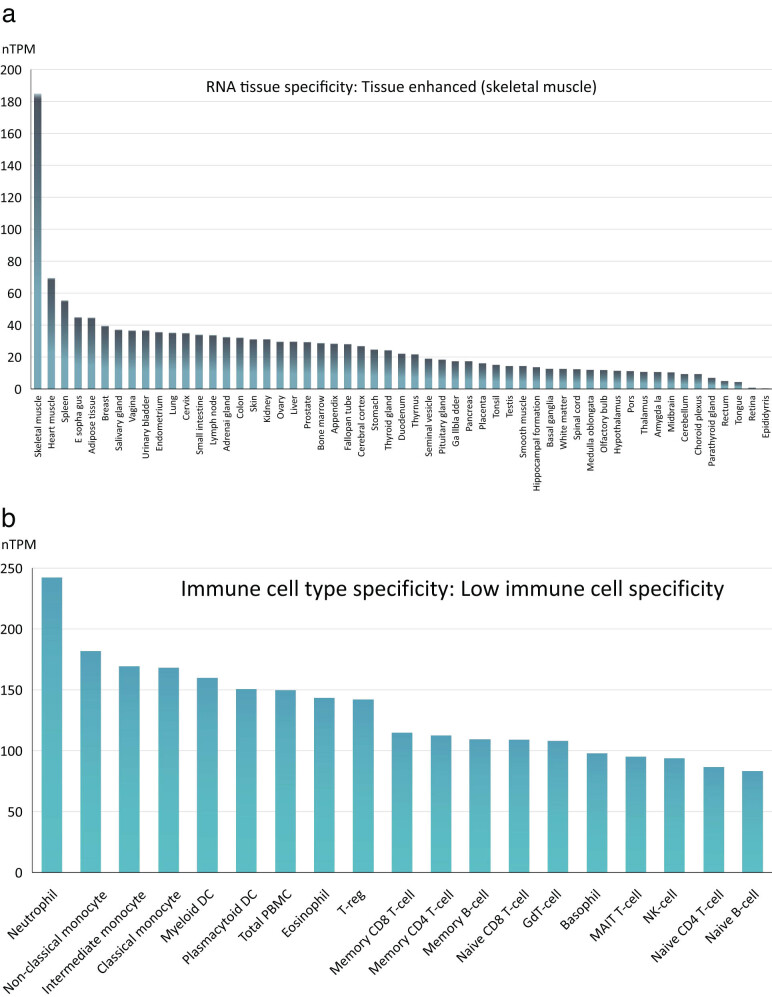
TWF2 transcription level in various cells, organs, and plasma during a healthy state of being. (a) Based on GTEx, HPA, and FANTOM5 databases, TWF2 gene transcription in various tissues; (b) Based on the Schmiedel, Monaco, HPA databases, we have determined the transcription of the TWF2 gene in several blood cell types.

The transcription patterns of TWF2 in various cell lines and non-tumor tissues were investigated in greater depth. We constructed our model based on the GTEx and HPA datasets, as shown in Figure A2a. This comparison revealed that TWF2 was substantially expressed, mainly in the skeletal muscle, granulocytes, and monocytes examined in this study. Analysis of the HPA datasets revealed that neutrophils had the highest TWF2 expression, followed by non-classical monocyte (Figure A2b).

**Figure 1:**
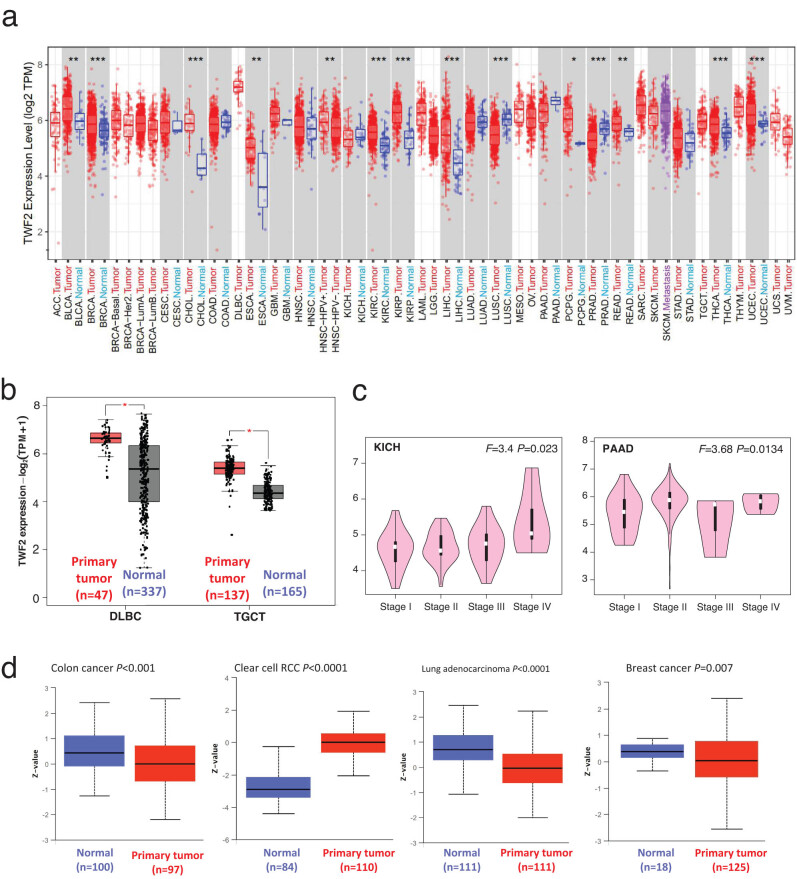
In human malignancies, TWF2 transcription and protein levels are elevated. (a) TWF2 transcription level in TCGA cancers compared to adjacent tissues (if available) shown using TIMER2. (b) Comparing TWF2 transcription level in DLBC and TGCT (TCGA project) to the equivalent healthy tissues using a box plot (GTEx dataset). (c) TWF2 transcription levels vary according to the stage. Using TCGA data, we analyzed and compared the major pathogenic stages of KICH, and PAAD, THCA, and UCS. Log2 (TPM + 1) transcription level are shown. (d) TWF2 total protein level in normal tissue and primary colon cancer, clear cell renal cell carcinoma, LUAD, and breast cancer. CPTAC was utilized to extract and evaluate protein data.

Using TIMER2, we then examined the transcription of TWF2 in tumors and adjacent normal tissues from TCGA datasets. This revealed a significant difference in the transcription between the two tissues. According to Figure 1a, the transcription level for the transcription factor TWF2 in the tumor tissues of UCEC, LIHC, KIRC, BRCA, CHOL, KIRP, and THCA with *p* < 0.001, READ, BLCA, and ESCA, with *p* < 0.01, that PCPG, with a *p* < 0.05, is higher than that of the corresponding control tissues. This was not the case with LUSC and PRAD, which had significantly reduced levels of TWF2 compared to control tissues (*p* < 0.001). TWF2 transcription variations between tumor and non-tumor tissues were further examined using the GTEx dataset in cases where TCGA data were unavailable. Both TGCT and DLBC had a higher level of transcription in tumor tissues than expected (Figure 1b, *p* < 0.05). According to the research, TWF2 is overexpressed in the vast majority of human malignancies. A correlation between higher TWF2 transcription and advanced tumor pathological staging was found using the GEPIA2 program in KICH and PAAD (Figure 1c, all *p* < 0.05).

The large-scale proteome program from the CPTAC (National Cancer Institute’s Clinical Proteomic Tumor Analysis Consortium) enabled us to analyze TWF2 at the protein level, in addition to its transcription. When TWF2 expression in clear cell RCC was compared to that in healthy tissues, we determined that it was much higher than that in healthy tissues, whereas it was much lower in colon cancer, LUAD, and breast cancer tissues (Figure 1d; *p* < 0.01).

### 3.2 Survival analysis

**Figure 2:**
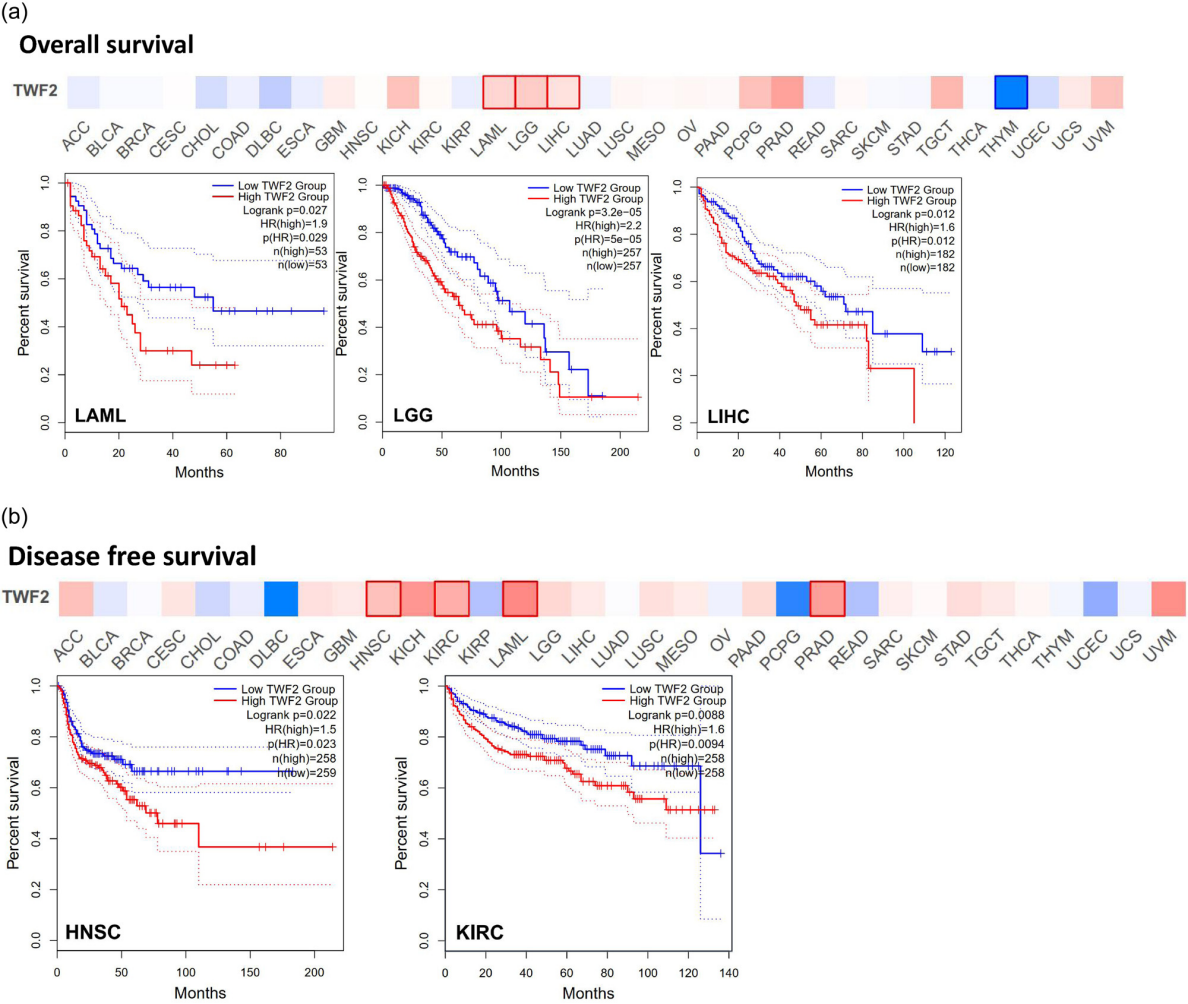
The correlation between the degree of TWF2 transcription and patient survival in TCGA tumors. The correlation between TWF2 gene transcription and OS (a), DFS (b). GEPIA2 was utilized to examine all of the tumors from the TCGA. The following table contains the favorable results of the survival map and Kaplan Meier curves.

The next step was to examine whether the presence of TWF2 was associated with a better prognosis or longer OS. To divide the cancer cases into high- and low-transcription groups, the TWF2 transcription level was measured. Then, using data from TCGA and GEO datasets, we investigated the relationship between TWF2 transcription and the prognosis of various tumor patients. TWF2 transcription was associated with poorer OS in several forms of cancer, including LAML (*p* = 0.03), LGG (*p* < 0.0001), and LIHC (*p* = 0.01) (Figure 2a). In the DFS study, TWF2 overexpression was associated with a poorer prognosis in HNSC (*p* = 0.02) and KIRC (*p* < 0.01) (Figure 2b).

**Figure 3:**
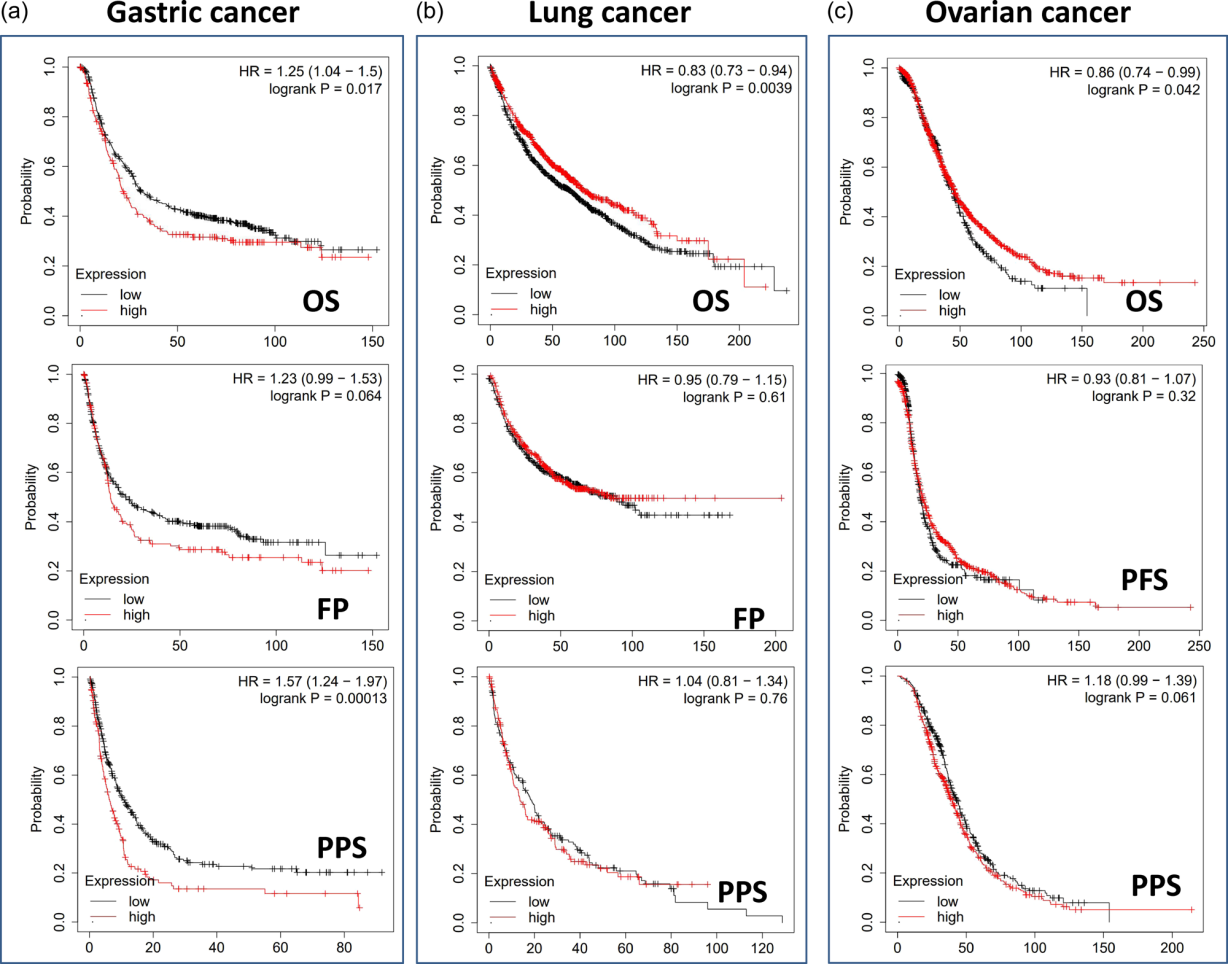
Analysis of TWF2 gene transcription and tumor prognosis using Kaplan–Meier plotting. It was decided to utilize a Kaplan Meier plotter to perform the survival studies in gastric cancer (a), lung cancer (b), and ovarian cancer (c) patients.

When we utilized the Kaplan–Meier plotter datasets to analyze the survival data, we discovered a correlation between high TWF2 transcription levels and poorer OS and PPS for gastrointestinal cancer. In contrast, we discovered a statistically significant difference between high TWF2 transcription levels and improved OS for lung and ovarian cancers (Figure 3).

### 3.3 Genetic alterations analysis

**Figure 4:**
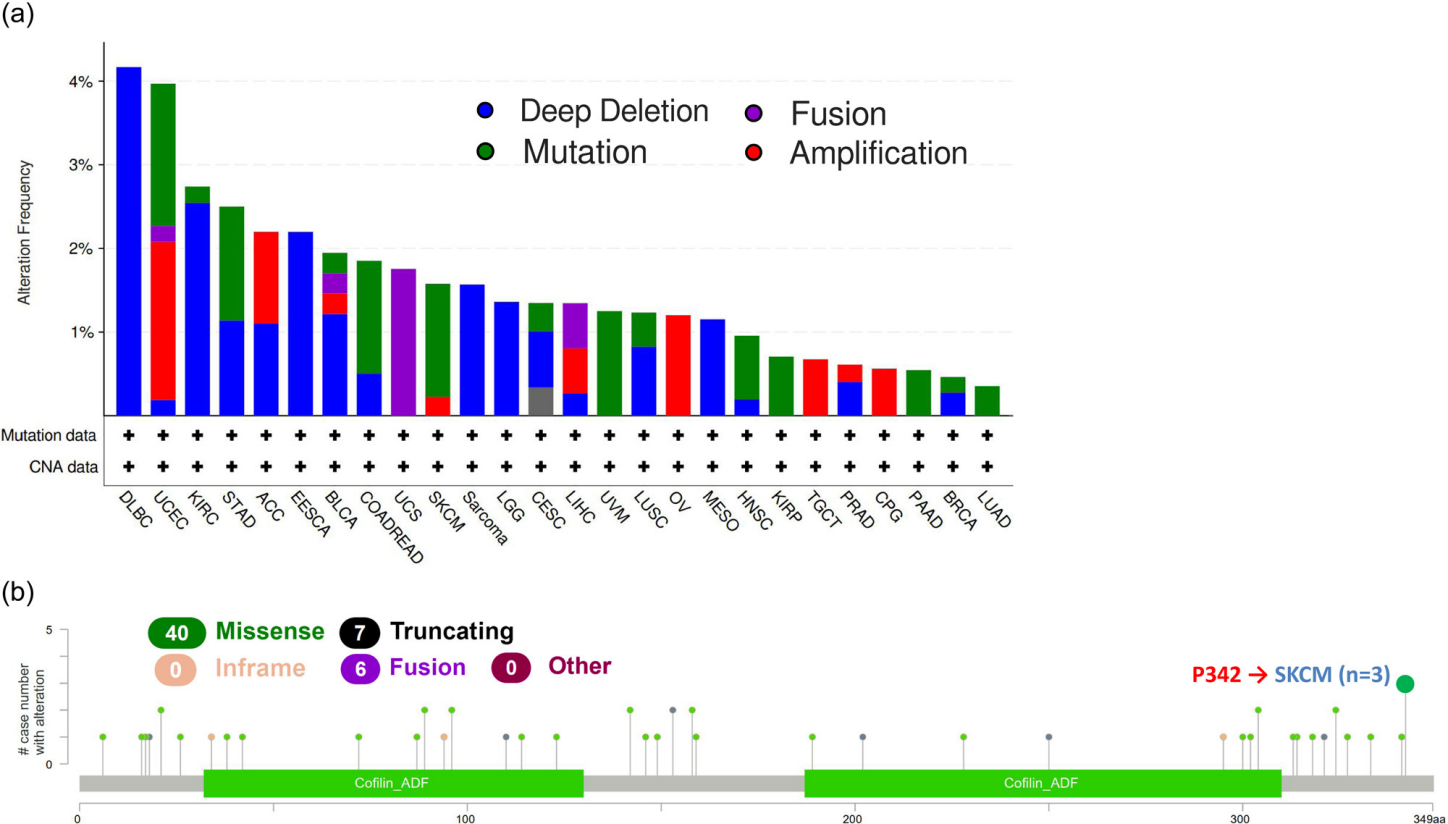
Mutation status of TWF2 in TCGA tumors. An analysis of the TWF2 mutation status in TCGA tumors was carried out using the cBioPortal program. The alteration frequency with mutation type (a), and mutation site (b).

Mutations in the human genome are the root cause of nearly all human malignancies. Therefore, we decided to investigate TWF2 genetic changes in human tumor tissues. According to our analysis, the frequency of TWF2 alteration (>4%) is highest in DLBC with “deep deletion” as the predominant kind of alteration. The “amplification” kind of Copy Number Alteration was most common in UCEC, occurring at a frequency of ∼2%. In addition, we noted that “deep deletion” of TWF2 in tumors frequently occurs in almost all cancer types (Figure 4a). Figure 4b further depicts the types, sites, and case numbers of the TWF2 genetic alteration. We found that missense mutation of TWF2 was the main type of gene alteration and P342 alteration, which was detected in three cases of SKCM (Figure 4b).

**Figure 5:**
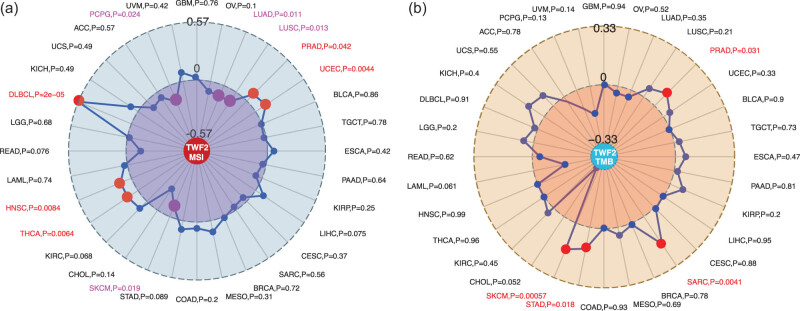
Correlation between MSI and TMB, and the transcription of TWF2, respectively. Data from the TCGA project was utilized to perform a correlation analysis between TWF2 transcription and MSI and TMB. There is a *p* value and partial correlation values of +0.57, −0.57, +0.33, and −0.33 respectively, shown in the graph.

Using TCGA tumor samples, we examined the correlation between the transcription of TWF2, MSI, and TMB. According to our findings, TWF2 transcription was positively correlated with MSI in PRAD, UCEC, THCA, HNSC, and DLBC (all *p* < 0.05) but was negatively correlated with LUAD, LUSC, SKCM, and PCPG (all *p* < 0.05) (Figure 5a). Additionally, TWF2 transcription in TMB was associated with STAD, SARC, PRAD, and SKCM (all *p* < 0.05) (Figure 5b).

### 3.4 Immune infiltration analysis

**Figure 6:**
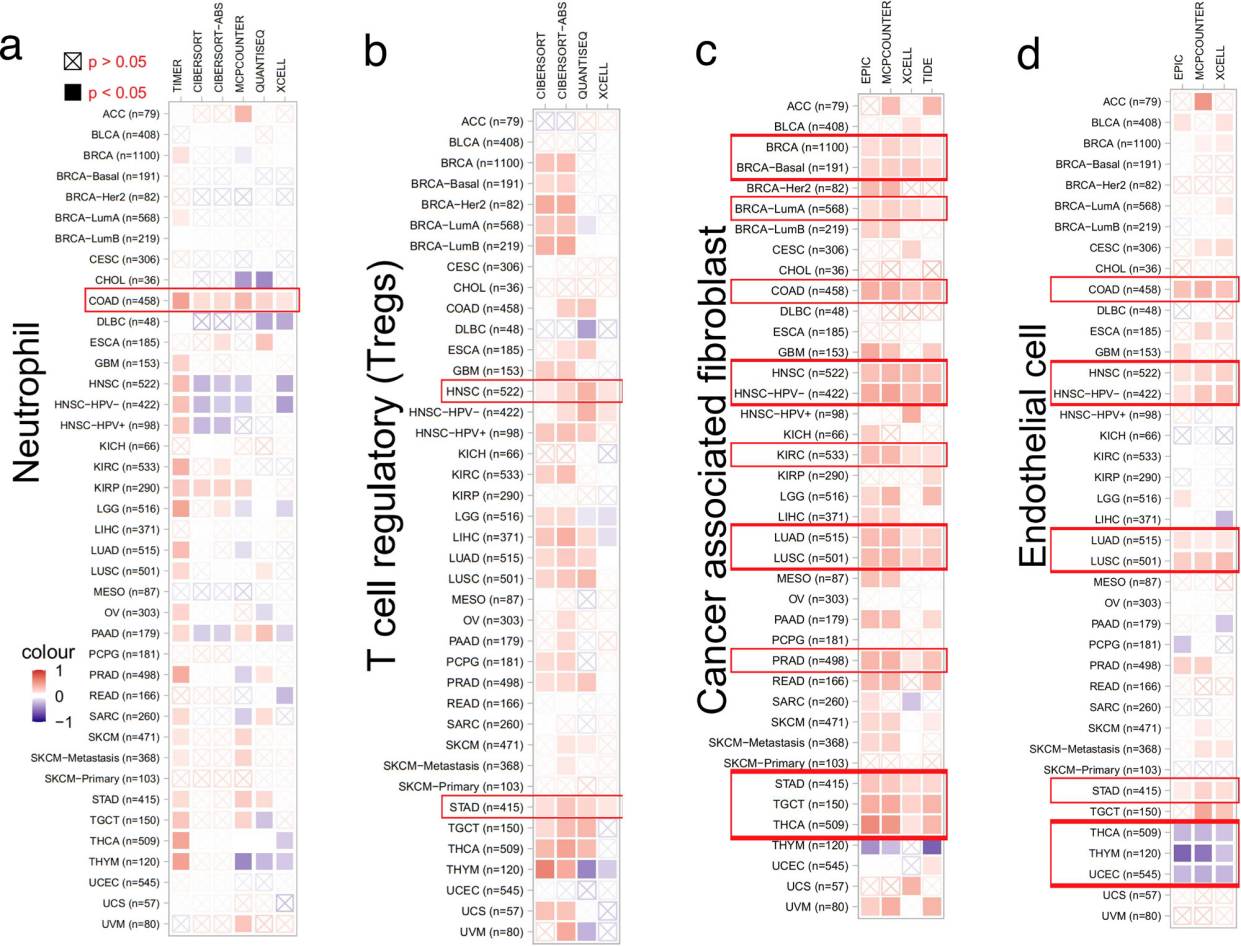
The association between the level of TWF2 transcription and the infiltration of cancer-related neutrophils, T-cell regulatory, cancer-associated fibroblast, and endothelial cells has been demonstrated. (a–d) TIMER, CIBERSORT-ABS, CIBERSORT, QUANTISEQ, EPIC, MCPCOUNTER, XCELL, and TIDE algorithms were utilized for analysis of the relationship between the infiltration amount of Neutrophil, T-cell regulatory, malignancy-related fibroblasts, the endothelial cell infiltration and the transcription level of TWF2 gene in all TCGA-tumors. The blue color denotes a negative correlation (−1∼0), and the red color denotes a positive correlation (0–1). The correlation with *p* < 0.05 is deemed important statistically. Statistically unessential correlations parameters are denoted by a cross. The red rectangle represents a consistent result assessed by different algorithms.

We anticipated that changing TWF2 transcript level or genetic changes in TWF2 would influence the tumor-infiltrating immune cell reaction because of the established role of the actin cytoskeleton in cell migration pathways and the involvement of TWF2 in the regulation of actin cytoskeleton structure [17,18,19]. As the results shown in Figure 6, we utilized the TIMER2 program to investigate the link between the infiltration of distinct endothelial and immune cells and TWF2 transcription in different tumor types from TCGA. Intriguingly, we found a positive association between TWF2 transcription and the predicted neutrophil infiltration value in COAD (Figure 6a). There is a positive association between TWF2 transcription and T-cell regulatory in HNSC and STAD (Figure 6b); cancer-associated fibroblasts for BRCA, BRCA-Basal, BRCA-LumA, COAD, HNSC, HNSC-HPV (−), KIRC, LUAD, LUSC, PRAD, STAD, TGCT, and THCA (Figure 6c); and endothelial cell infiltration for COAD, HNSC, HNSC-HPV (−), LUAD, LUSC, and STAD. TWF2 transcription was negatively correlated with endothelial cells in THCA, THYM, and UCEC (Figure 6d).

### 3.5 TWF2 partner enrichment analysis

**Figure 7:**
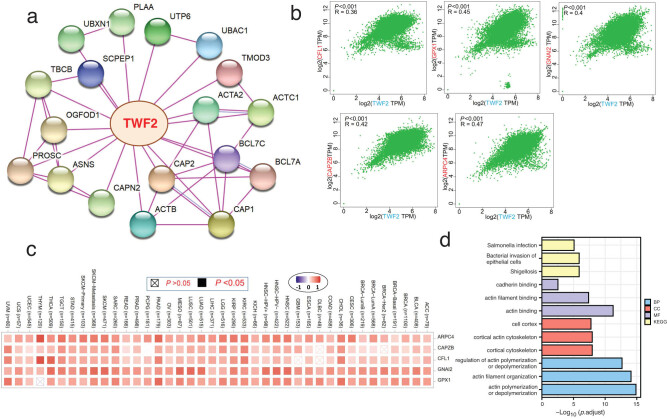
TWF2 associated gene enrichment and pathway analysis. (a) Experimentally verified TWF2-binding proteins are shown as a STRING protein network map. Identified proteins are represented as colored nodes on the graph. (b) correlation between TWF2 transcription and representative genes (CFL1, GPX1, GNAI2, CAPZB, and ARPC4) from the top TWF2 co-related genes in the TCGA studies as identified by GEPIA-2. (c) TCGA tumors were utilized to generate a heatmap depiction of the data on the transcription correlations between TWF2 and the genes CFL1, GPX1, GNAI2, CAPZB, and ARPC4 in the tumors. (d) The TWF2-binding and associated genes were examined in an intersection analysis. (e) TWF2-binding and interconnected genes, GO/KEGG pathway-based analysis.

The penultimate phase in our inquiry into the molecular mechanism of the TWF2 gene in cancer and development was to filter out all recognized TWF2-interacting proteins and TWF2 expression-correlated genes, which we did as the last step. STRING allowed us to identify a total of 50 interacted TWF2 proteins that had been previously identified. Figure 7a depicts the interaction network of 18 proteins. TWF2 was shown to be associated with the transcription of the top 100 genes that had been combined from TCGA tumor transcription data. TWF2 transcription was found to be positively linked with the transcription of CFL1 (*R* = 0.36), GPX1 (*R* = 0.45), GNAI2 (*R* = 0.4), CAPZB (*R* = 0.42), and ARPC4 (*R* = 0.47) genes (all *p* < 0.001) (Figure 7b). TWF2 was found to have a high positive connection with the five genes listed above in the majority of tumor types, according to heatmap data (Figure 7c). We integrated the two databases and performed GO and KEGG enrichment studies on the combined results. A search for GO|KEGG pathways found that “shigellosis,” “actin binding,” “cortical cytoskeleton,” and “acting polymerization or depolymerization” were among the top hits, indicating that the influence of TWF2 on tumor etiology maybe mediated through these pathways (Figure 7d).

## 4 Discussion

It is unclear whether TWF2 is involved in the oncogenesis of specific tumor types or whether it is involved in more general pathways that contribute to tumor pathogenesis. Therefore, we conducted a TWF2 pan-cancer analysis in this study. So far, there have been few studies related to TWF2 in the field of cancer research. When CHAF1B was knocked down, the protein and mRNA levels of TWF2 were considerably reduced in the human hepatocellular carcinoma cell line HUH-7, thus decreasing the invasion and migration of the tumor [20].

We utilized TCGA and GEO datasets and various bioinformatics techniques to investigate the oncogenic role of TWF2 in this study. The data from the “HomoloGene” research revealed the conservation of the TWF2 protein structure across species. TWF2 transcription was shown to be higher in cancerous tissues than in normal tissues in a study comparing the two types of tissues. According to survival analysis, TWF2 transcription was found to be associated with poor prognosis in various forms of cancer. Genomic changes in TWF2 have been observed in tumor samples spanning a variety of cancer types, including deletions, amplifications, and mutations. TWF2 transcription, MSI, and tumor mutational burden have been found to be positively associated with various cancers.

TWF2 transcription in tumor samples from patients was higher in UCEC, THCA, READ, PCPG, LIHC, KIRP, KIRC, ESCA, CHOL, BRCA, and BLCA tumors than in the control samples. This was not the case in our study of samples from patients with LUSC and PRAD, which had lower levels of TWF2 expression. Depending on the type of tumor, the TWF2 transcription level can vary significantly. This could be due to its distinct functions and mechanisms in different tumors. Both TWF1 and TWF2 had a significantly higher expression in UCEC, LIHC, BRCA, CHOL, KIRP, THCA, and ESCA tissues [11]. TWF2 overexpression was also reported to be related to poorer prognosis in patients with cancers such as LAML, LGG, LIHC, HNSC, and KIRC, which express a high level of TWF2. Thus, TWF2 may be one of an indicator for cancer patients’ prognosis, which is supported by these findings.

In contrast, our previous research of TWF1 [11] showed a quite different mutation profile. The frequency of TWF1 alteration (>5%) is the highest in uterine tumors with “mutation” as the primary type. ACC had the highest incidence of “amplification” type of CNA, with a frequency of ∼4%. TWF1 “amplification” kind in tumors frequently occurs in almost all tumors. This may be caused by the structural differences and tissue distribution differences of TWF1 and TWF2 gene subtypes, and the genetic alteration differences may explain the functional differences of TWF1 and TWF2 proteins and the differences in the expression and regulation of cell signaling pathways [7].

In this study, TWF2 transcription, MSI, and TMB were linked. Our data showed that TWF2 transcription is positively correlated with the number of endothelial and tumor-associated fibroblast cells that have been deconvolved using several immunodeconvolution methods. TWF2 was positively associated with cancer-associated fibroblasts in STAD and TGCT and with endothelial cells in LUAD, LUSC, and STAD. However, the relationship between TWF1 and corresponding immune cells in the above tumors was largely opposite [11]. This may mean that there are differences in the roles of TWF1 and TWF2 in the immune microenvironment in these tumors.

Despite the fact that our research generated helpful results, we recognize that it has several limitations. First, with further studying, the relationship between TWF2 and tumor may become clearer and closer. Relevant results are constantly updated, so the results presented in this study may not be comprehensive. Second, a large number of experiments need to be validated and further explored.

TWF2 transcription was found to be statistically associated with clinical prognosis, immune cell infiltration, MSI, and tumor mutation burden in a range of human malignancies, helping to clarify the role of TWF2 in carcinogenesis from various perspectives.

**Table 1:** Abbreviation list

Abbreviation	Definition
ACC	Adrenocortical carcinoma
BLCA	Bladder urothelial carcinoma
BRCA	Breast invasive carcinoma
CESC	Cervical squamous cell carcinoma and endocervical adenocarcinoma
CHOL	Cholangiocarcinoma
COAD	Colon adenocarcinoma
READ	Rectum adenocarcinoma
DLBC	Lymphoid neoplasm diffuse large B-cell lymphoma
ESCA	Esophageal carcinoma
GBM	Glioblastoma multiforme
HNSC	Head and neck squamous cell carcinoma
KICH	Kidney chromophobe
KIRC	Kidney renal clear cell carcinoma
KIRP	Kidney renal papillary cell carcinoma
LAML	Acute myeloid leukemia
LGG	Brain lower grade glioma
LIHC	Liver hepatocellular carcinoma
LUAD	Lung adenocarcinoma
LUSC	Lung squamous cell carcinoma
MESO	Mesothelioma
OV	Ovarian serous cystadenocarcinoma
PAAD	Pancreatic adenocarcinoma
PCPG	Pheochromocytoma and paraganglioma
PRAD	Prostate adenocarcinoma
READ	Rectum adenocarcinoma
SARC	Sarcoma
SKCM	Skin cutaneous melanoma
STAD	Stomach adenocarcinoma
TGCT	Testicular germ cell tumors
THCA	Thyroid carcinoma
THYM	Thymoma
UCEC	Uterine corpus endometrial carcinoma
UCS	Uterine carcinosarcoma
UVM	Uveal melanoma
